# The Epidemiology and Variation in Pseudorabies Virus: A Continuing Challenge to Pigs and Humans

**DOI:** 10.3390/v14071463

**Published:** 2022-07-01

**Authors:** Qingyun Liu, Yan Kuang, Yafei Li, Huihui Guo, Chuyue Zhou, Shibang Guo, Chen Tan, Bin Wu, Huanchun Chen, Xiangru Wang

**Affiliations:** 1 State Key Laboratory of Agricultural Microbiology, College of Veterinary Medicine, Huazhong Agricultural University, Wuhan 430070, China; lqy948987886@163.com (Q.L.); ky19981459286@163.com (Y.K.); lyfovlyf@163.com (Y.L.); guohuihui0216@163.com (H.G.); dyp053224@163.com (C.Z.); shibang_guo@163.com (S.G.); tanchen@mail.hzau.edu.cn (C.T.); wub@mail.hzau.edu.cn (B.W.); 2 Key Laboratory of Preventive Veterinary Medicine in Hubei Province, The Cooperative Innovation Center for Sustainable Pig Production, Wuhan 430070, China; 3 Key Laboratory of Development of Veterinary Diagnostic Products, Ministry of Agriculture of the People’s Republic of China, Wuhan 430070, China; 4 International Research Center for Animal Disease, Ministry of Science and Technology of the People’s Republic of China, Wuhan 430070, China

**Keywords:** pseudorabies virus, epidemiology, variation, pig, human pseudorabies encephalitis

## Abstract

Pseudorabies virus (PRV) can infect most mammals and is well known for causing substantial economic losses in the pig industry. In addition to pigs, PRV infection usually leads to severe itching, central nervous system dysfunction, and 100% mortality in its non-natural hosts. It should be noted that increasing human cases of PRV infection have been reported in China since 2017, and these patients have generally suffered from nervous system damage and even death. Here, we reviewed the current prevalence and variation in PRV worldwide as well as the PRV-caused infections in animals and humans, and briefly summarized the vaccines and diagnostic methods used for pseudorabies control. Most countries, including China, have control programs in place for pseudorabies in domestic pigs, and thus, the disease is on the decline; however, PRV is still globally epizootic and an important pathogen for pigs. In countries where pseudorabies in domestic pigs have already been eliminated, the risk of PRV transmission by infected wild animals should be estimated and prevented. As a member of the alphaherpesviruses, PRV showed protein-coding variation that was relatively higher than that of herpes simplex virus-1 (HSV-1) and varicella-zoster virus (VZV), and its evolution was mainly contributed to by the frequent recombination observed between different genotypes or within the clade. Recombination events have promoted the generation of new variants, such as the variant strains resulting in the outbreak of pseudorabies in pigs in China, 2011. There have been 25 cases of PRV infections in humans reported in China since 2017, and they were considered to be infected by PRV variant strains. Although PRV infections have been sporadically reported in humans, their causal association remains to be determined. This review provided the latest epidemiological information on PRV for the better understanding, prevention, and treatment of pseudorabies.

## 1. Introduction

Pseudorabies virus (PRV), the causative agent for Aujeszky’s disease, belongs to the family Herpesviridae, subfamily Alphaherpesvirinae, and genus Varicellovirus [1]. Similar to other members of the Varicellovirus, PRV is neurotropic and can establish latent infection in the peripheral nervous system [2,3]. Pigs are the natural hosts of PRV, showing neurological disorders in newborn piglets and reproductive failure in sows after infection [4]. Worldwide attempts to control PRV infection in pigs have been ongoing for decades by attenuated marker vaccines with virulence-associated gene deletion and respective serological diagnostic tests [5]. However, long-term immune pressure could promote PRV variation for immune escape, creating new challenges for the future prevention and control of pseudorabies. Moreover, PRV infection in humans has been reported recently, and the number of cases has been increasing since 2017, but the causative association and the pathogenic mechanism remain unclear. In addition to its pathogenicity, PRV has been widely studied as an ideal model for investigating herpesviruses’ molecular biology and pathogenic mechanism [6]. It has also been utilized as a living tracer in neural circuits and a promising oncolytic virus [7]. Therefore, it is of significant importance to understand the current clinical prevalence and variation in PRV for the better understanding, prevention, and control of pseudorabies and the appropriate application of PRV.

## 2. Epidemiology of PRV

### 2.1. The Prevalence of PRV in the World

**Figure 1:**
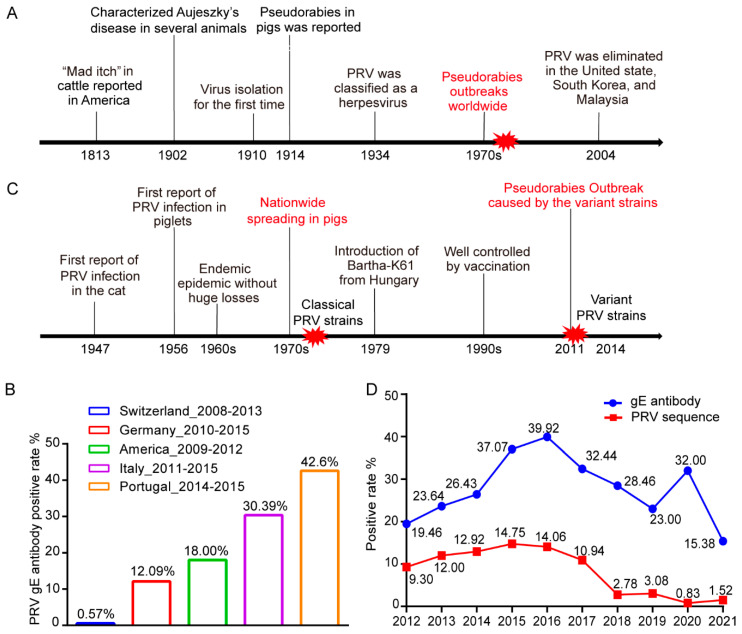
The prevalence of pseudorabies worldwide: (**A**) epidemic history of PRV worldwide. The red explosion shape represents outbreaks of pseudorabies. (**B**) The reported surveillance of PRV infection in wild boars, as illustrated by PRV gE antibody positive rate. (**C**) Epidemic history of PRV in China. The red explosion shape represents outbreaks of pseudorabies. (**D**) The positivity rate of PRV gE antibody and PRV *gE* nucleotide sequences detected in nationwide samples in China from 2012 to 2019.

PRV infection was first defined as “mad itch” in bovines in America in 1813 [8], and PRV was successfully isolated about 100 years later [9]. With the global development of the pig industry, pseudorabies caused by PRV firstly broke out in pigs worldwide during the 1970s–1980s and was a pandemic for decades (Figure 1A). Currently, PRV is mainly circulating in domestic pigs in Argentina, Bosnia and Herzegovina, China, Croatia, Cuba, France, Hungary, Italy, Mexico, Papua New Guinea, Poland, Portugal, Spain, and the United States of America, according to OIE reports from 2019 to 2021 [10]. Due to efficient vaccination and eradication measures, pseudorabies in domestic pigs has been eliminated in Germany, the United Kingdom, Ireland, South Korea, Sweden, Colombia, Denmark, New Zealand, and many other countries. However, it is difficult to maintain the elimination status, as indicated by second outbreaks of pseudorabies in Argentina in 2019 and France and Mexico in 2020 [10].

In the countries or districts in which pseudorabies has eliminated in domestic pigs, virus transmission from infected wild boars is a critical threat for these domestic pigs. Therefore, serological investigations in wild boars have been conducted in many countries to monitor the transmission risk (Figure 1B). In Italy, the PRV prevalence in wild boars varied from 4% to 30% because of the different densities of wild boar populations, and 30.39% of 1425 sera samples collected from wild boars between 2011 and 2015 in northwest Italy were positive for PRV antibodies [11]. An overall nationwide PRV seroprevalence of 12.09% was detected from 108,748 sera samples from wild boars in Germany from 2010 to 2015 [12]. The PRV seroprevalence rate of wild boars in Switzerland is the lowest among those recorded in Europe, with samples collected between 2008 and 2013 having a seroprevalence of 0.57% [13]. In the United States, 8498 sera samples were collected from wild boars in 35 states from 2009 to 2012, among which the samples from 25 states had a total positivity rate of 18% [14]. The above data indicate a high prevalence of PRV in wild boars and a risk of transmission to domestic pigs. Therefore, routine measures, including fencing and disinfection, should be taken in the epizootic areas with pseudorabies to prevent direct transmission from contact between wild boars and domestic pigs or indirect transmission mediated by people and hunting tools. Moreover, it has been proposed to reduce PRV prevalence in wild boars by controlling the density of wild boars [11] and the reactivation and spillover of latent PRV. In summary, for the pig farms in most countries, it is essential to ensure a sufficient biosafety distance between domestic pigs and wild boars and to ensure appropriate control of pseudorabies prevalence in wild boars.

### 2.2. The Prevalence of PRV in China

Pig farms in China have suffered from large-scale outbreaks of pseudorabies since the 1970s (Figure 1C). The natural attenuated vaccine strain Bartha-K61 was imported in 1979, and several attenuated strains developed from local classical PRV strains such as Ea and Fa were also utilized to control the pandemic, leading to a remarkably reduced prevalence after 1990. However, by the end of 2011, another PRV outbreak occurred from variant PRV strains, even in the pig farms with routine immunization. Since then, the PRV prevalence rate in China has raised sharply and remains high in some provinces (Table 1).

PRV *gE* sequences and antibodies in samples collected nationwide from 2012 to 2021 were detected to monitor the prevalence of pseudorabies in pigs in China. Since the commercial PRV vaccines are all strains with *gE* gene deletion, the gE antibody is considered to be an indicator of infection caused by wild strains. Additionally, the detection of the *gE* sequence indicates the presence of the virus in pigs. gE antibody prevalence has increased rapidly since the occurrence of variant strains in 2011, and it peaked at 39.92% (3733/9350) in 2016, when the positive rate of *gE* specific sequence was as high as 14.06% (399/2837). Subsequently, the PRV gE antibody and sequence positivity rate gradually decreased to 15.38% (5971/38,821) and 1.52% (53/3503) in 2021, respectively, probably attributed to by the development and application of vaccines based on the variant strains (Figure 1D). The updated variant vaccine strains and the decreased prevalence supported the importance of the high genomic identity between vaccine strains and field strains. However, several provinces in China still show a serious epidemic situation of pseudorabies, with gE antibody prevalence varying from 7.50% to 62.74% (Table 1). Although pseudorabies in domestic pigs in China is currently under control, it is necessary to monitor the variation in PRV strains and to accelerate the current elimination programs.

## 3. Genotyping and Variation in PRV

### 3.1. Genotyping of PRV

Different PRV strains differ in biological characteristics even though they are in one serotype. The restriction fragment length pattern (RFLP) was used in PRV genotyping [29], especially in RFLP based on B*amH*I. B*amH*I-RFLP divides the PRV strains into genotypes I-IV [30,31,32]. B*amH*I-mPCR is a method that combines B*amH*I-RFLP with the highly sensitive multiplex PCR. It can be applied to PRV genotyping in samples with a low DNA content without virus isolation [33]. Genotyping based on the *gC* gene and genomes has been increasingly applied in the development of sequencing technology. The *gC* gene is one of the most variable regions in the genome [34]. Based on the phylogenetic analysis of 729 global *gC* sequences, PRV can be divided into two genotypes with Chinese isolates in genotype II and with isolates from other places in genotype I. The most recent common ancestor of the two genotypes was divided into two genotypes and evolved separately around A.D. 1013 [35]. PRV strains in genotype I can be divided into six subtypes, and subtype 1.6 includes Chinese isolates that are closely related to Bartha-k61 [35]. Genotype II can be divided into two subtypes. Subtype 2.1 contains Chinese classical strains isolated in the 1990s, and subtype 2.2 mainly consists of the variant strains isolated after 2011 [36]. In addition, tandem short sequence repeats (SSRs), a class of nucleic acids motifs, might be another molecular basis for PRV genotyping in future studies. SSRs exist in almost 20% of the PRV genome. The changes in length in the SSRs have been associated with DNA binding site efficiency, transcription regulation, and protein interactions [37]. Therefore, the differences in SSR length between strains might explain the differences in the biological characteristics of different PRV strains in the same serotype.

### 3.2. The Evolution of PRV Based on Natural Mutation-Selection

Alphaherpesvirinae genomes are relatively stable with minor variation in the sequences among strains. The average rate of protein-coding variation in PRV was 1.6%, which is higher than the 1.3% of herpes simplex virus-1 (HSV-1) and the 0.2% of varicella-zoster virus (VZV) [37]. The mean substitution rate of the PRV genome is 4.82 × 10^−5^ substitutions per site annually [35]. Furthermore, Bayesian skyline coalescent reconstruction illustrated that the relative genetic diversity of genotype I remained unchanged, while in genotype II, the diversity decreased from 2004 to 2010 and increased sharply from 2010 to mid-2012 and was maintained at a high level in 2016 [36]. The time points of the diversity changes in genotype II are consistent with those of pseudorabies control and epidemic in China.

Natural mutation-selection could contribute to the diversity changes in the PRV strains in genotype II. Positive selection has been detected in the amino acid residues at site 43/75/505/834/848/908/922 of gB, site 348/575/578 of gE, and site 59/75/194 of gC but not in gD [36,38,39]. In addition, site 929/934 of gB, site 495/540 of gE, and site 59/75/76/191 of gC are involved in the adaptive evolution after cross-species transmission. The amino acid residue at site 59 of gC participates in positive selection and adaptive evolution, the function of which is related to the viral adsorption process [36]. The variation in the gB, gE, and gC proteins in Chinese variants of PRV may facilitate escaping from the host immune response and adapting to the new host after cross-species transmission.

The genetic diversity supported by SSRs might also promote PRV evolution. SSRs have been found in all herpesviruses. Their length varies in different strains of PRV and HSV-1, and a few SSRs diversity can be detected, even during the PRV plaque purification. In the SSR analysis of Kaplan, Becker, Bartha, and other strains, it was observed that SSRs existed in both coding and non-coding sequences, promoters, and open intergenic sequences, mainly in the IR-US-TR region. Furthermore, 62% of the SSRs in PRV, including most of the SSRs in the coding region, contain triplet-based repeats, such as 3-mer, 9-mer, 27-mer, etc. These triplet-based SSRs not only contribute to genetic diversity but also remain the original frame of the coding sequence [37]. These subtle changes, such as changes in SNPs and SSR length, support genetic diversity and promote PRV evolution.

### 3.3. Frequent Recombination between PRV Strains Significantly Contributes to Virus Evolution

The frequent inter- and intra-genotype recombination of PRV has been reported (Table 2). Recombination between the field strains is important for PRV evolution since alphaherpesviruses have DNA polymerases with high proof-reading activity and exonuclease activity [40]. There was a high recombination rate in vivo after co-inoculating different PRV strains in sheep and pigs [41,42]. In another report, a South Korean isolate (Yangsan) was located between genotype I and genotype II in the phylogenetic tree base on UL21 when located in genotype II in the phylogenetic trees based on US2, gD, and US9, which suggested recombination between genotypes I and II in UL21 [35]. Similarly, inter-clade recombination between genotypes I and II was detected in gB of PRV FJ-W2, FJ-ZXF, and FJ62 [38,43]. There was a recombination analysis of 29 full-length genomes, and more than four of the seven methods showed that almost all of the PRV strains demonstrated recombination. It was suggested that intra-clade recombination was more frequent than inter-clade recombination. Moreover, Chinese variant strains such as HeN1 and Qihe547 may have originated from the recombination between the isolates in genotype I and the vaccine isolates in genotype II (such as Ea and Fa) [36].

In addition, recombination between the field isolates and vaccine strain Bartha-K61 has been frequently detected. JSY13, which was isolated in Jiangsu in 2018, has been found to be a natural recombinant strain between Bartha-K61 in genotype I and JSY7 in genotype II. The recombination involves the genes *UL42*, *UL19*, *UL18*, and *UL10* [44]. Moreover, the earlier isolates in genotype II such as SC and LA may have originated from recombination between the foreign isolates (such as Bartha-K61) and the early epidemic PRV in China [36]. Consistently, it has been reported that SC is a recombinant strain between the Chinese early local PRV isolate and the vaccine strain Bartha-K61 [45]. In our recombination analysis based on 55 PRV genomes, a total of 23 recombination events were identified, with 16 events observed between Bartha-K61 and the Chinese strains [39]. The vaccine strain Bartha-K61 has been widely used to control porcine pseudorabies in China for decades. These recombination events, especially those between vaccine strains and field strains, suggest that long-term immunity has dramatically contributed to the variation and evolution of PRV, which may explain the pseudorabies variant outbreak in China in late 2011.

## 4. PRV Infections in Animals and Potentially in Humans

### 4.1. PRV Infections in Pigs and Other Animals

Pigs are the natural host and reservoir of PRV. Infected newborn piglets can show neurological symptoms on the second day after birth, including screaming, ataxia, opisthotonos, and padding, and mortality can be as high as 100%. In contrast, infected fattening pigs generally show temporary temperature elevation, respiratory symptoms, and low mortality with occasional neurological symptoms. Moreover, PRV infection causes severe reproductive disorders, including orchiditis and epididymitis in boars and pregnancy failure in sows [46,47]. Additionally, PRV can establish latency in the peripheral nervous system of the tolerated pigs after infection. Latent infection is characterized without virus replication and clinical symptoms. After latency, virus reactivation can be triggered by certain factors that interfere with host immunity [6], resulting in virus spillover and disease outbreaks. Thus, latency in pigs is a major risk and an obstacle in the late stage of PRV elimination. Future research on the virus latency is critical for establishing PRV-free domestic pig herds.

PRV is also infectious to many other mammals, including ruminants, carnivores, and rodents, and is characterized by severe itching and central nervous system (CNS) dysfunction with 100% mortality [48]. PRV infections in non-natural hosts are generally experimental infections or natural infections likely associated with pigs. Natural infections in farmed cattle have been reported worldwide and are related to contact with infected pigs [49,50]. Infected cattle show mad itch, epilepsy, and paralysis [51]. In 2018, nine cattle were infected by the Chinese variant PRV strain SDLY-China-2018. The infected cattle were raised very close to the pigs positive for the gE antibody, suggesting possible virus transmission from pigs to cattle [49]. In addition, an outbreak of pseudorabies was reported in a flock of 160 ewes housed next to PRV-infected pigs under virus spillover, and 5 cats on this farm were also infected by PRV [52]. Moreover, it has been observed that PRV cannot be horizontally transmitted between infected sheep and healthy sheep [53].

In companion animals, cats and dogs can be infected by PRV through contact with infected pigs, and hunting dogs are more susceptible due to frequent contact with wild animals [54,55]. These dogs died shortly after showing neurological symptoms [56].

PRV infections in wild animals have also been widely reported, including in wild boars [57], foxes [58], wolves [59], brown bears [60], black bears [61], Florida cheetahs [62], lynx [63], and raccoons [64]. In the experimental infection of raccoons and pigs, PRV transmission did not occur between raccoons but did occur between raccoons and swine via contact or predation [64], which was similar to the transmission pattern between sheep and pigs. In 2014, a mink farm in northern China suffered from PRV infection due to feeding raw pork contaminated PRV, resulting in diarrhea, neurologic signs, and 80–90% mortality [65].

According to the above virus transmission patterns of pig–cattle, pig–sheep, pig–cat, pig-dog, and wild boar–hunting dog, it is likely that pigs are the core reservoir for the PRV cross-species transmission. However, PRV infection in non-reservoir animals is different from that in pigs. Under natural infection conditions, these non-natural hosts develop itching, severe neurological symptoms, and even death, while there is no latent infection.

### 4.2. Potiential PRV Infections in Humans

It is controversial whether PRV can infect humans for the past one hundred years. No PRV-specific neutralizing antibody has been detected in 455 individuals with suspicious symptoms or occupations that put them at risk for infection, and no symptoms have been observed in volunteers injected with PRV at doses of 10^3.4^ TCID_50_ (intradermal) or 10^6.1^ TCID_50_ (subcutaneous) [66]. These results indicate that humans are not susceptible to PRV infection, or at least not to the PRV strain used. However, in 1914, two laboratory workers had their hands injured during contact with a PRV-infected cat and developed itching and swelling of the wound. As a result, PRV infection was suspected [66]. Moreover, three human cases have been reported in Europe, showing positive responses to PRV-specific neutralizing antibodies and neurological symptoms such as dysphagia, paresthesia, and tinnitus [67]. From 1914 to 1992, there were 17 reported cases of suspected PRV infection, and these patients developed pruritus, weakness, and pain (Table 3).

Since 2017, 25 more human cases of PRV infection have been reported. These cases were diagnosed by detecting PRV-specific antibodies with enzyme-linked immunosorbent assay (ELISA) and PRV nucleotides with PCR or metagenomic next-generation sequencing (Table 4). Notably, the PRV strain hSD-1/2019 was isolated from the cerebrospinal fluid sample of one patient, providing direct etiological evidence for PRV infection in humans [70]. Among the 25 cases, 100% of patients showed high fever and neurological symptoms; 56% showed severe visual impairment, including acute retinal necrosis, vitreous opacity, and blindness; and 16% of the patients died. In addition, 95% of the survivors suffered from severe sequelae, including visual impairment, vegetativeness, cognitive impairment, and memory loss. The CNS dysfunction related to PRV infection in these human cases has been defined as pseudorabies encephalitis (PRE) [71]. 

All 25 of these patients had a contact history with pigs or pork, indicating the importance of the infected pigs in human infection with PRV. However, more evidence is needed to support viral transmission from pigs to humans. Currently, it is believed that there are no reported cases of human–human transmission since the contacts of the patients have remained healthy. According to our investigation, the gB antibody-positive rates were 40.91% and 45.95% in the contacts of the two patients, while the gE antibodies were all negative [72]. Moreover, a retrospective investigation of 1335 serum samples from patients with encephalitis in 2012, 2013, and 2017 showed gB antibody positivity rates of 12.16%, 14.25%, and 6.52%, respectively [73]. Therefore, the positivity rates of the gB antibody in the associated populations were unable to be ignored, and gE antibody seroconversion could be an essential basis for diagnosis. 

**Table 4:** Case reports of human infection with PRV between 2017 and 2021.

Case	Year	Occupation	Contact History	Clinical Symptoms	Antibody Detection	Nucleotide	Outcome	Reference
1	2017	Swineherder	Sewage spilled into eyes	Fever, headache, visually impaired, endophthalmitis	gB antibody	+	Survived	[74]
2	2017	Pork dealer	Cut hand by a meat cleaver	Fever, headache, consciousness disorders, seizures, retinitis, encephalitis	PRV antibody-positive in three patients	+	Survived	[71]
3	2017	Cook	/	Fever, headache, seizures, consciousness disorders		+	Died	
4	2017	Pig butcher	/	Fever, headache, seizures, consciousness disorders		+	Survived	
5	2018	Pig butcher	/	Fever, seizures, consciousness disorders, retinitis		+	Survived	
6	2018	Veterinary	Hands were punctured by a knife used for the autopsy of dead swine	Fever, headache, seizures, respiratory failure, disturbance of consciousness, encephalitis	gB antibody gE antibody	+	Survived	[75]
7	2018	Swineherder	Needlestick injury	Fever, seizures, consciousness disorders, encephalitis	neutralizing antibody	+	Survived	[76]
8	2018	Pig butcher	Finger hurt by a pig	Fever, headache, visual disturbances, convulsions	/	+	Survived	[77]
9	2018	Pig butcher	Hand injury before hospitalization	Fever, memory loss, consciousness disorders, convulsions, respiratory failure		+	Survived	
10	2018	Swineherder	Hand injury before hospitalization	Fever, extremity tremors, respiratory failure, vision loss		+	Survived	
11	2018	Porker cutter	Hand injury at work	Fever, convulsions, respiratory failure		+	Survived	
12	2018	Porker cutter	No injury	Fever, extremity tremors, respiratory failure, vision loss		+	Survived	
13	2011	Pork dealer	/	Fever, psychotic behavior, seizures			Died	[78]
14	2018	Pig butcher	/	Fever, seizures, consciousness loss, retinal necrosis		+	Died	
15	2018	Swineherder	/	Fever, seizures, cognitive decline, respiratory failure, blindness		+	Survived	
16	2018	Driver	/	Fever, seizures, consciousness loss		+	Survived	
17	2019	Pork dealer	Contact with pork with injured fingers	Fever, seizures, consciousness disorder, encephalitis	PRV antibody positive	+	Survived	[79]
18	2018	Veterinary	/	Fever, headache, memory loss, seizures, consciousness disorders	gB antibody gE antibody Neutralizing antibody	+	Survived	[70]
19	2019	Pig butcher	Hand injury	Fever, headache, respiratory failure, memory loss, seizures, consciousness disorders		+	Survived	
20	2019	Pig butcher	Finger injury	Fever, headache, respiratory failure, memory loss, seizures, consciousness disorders		+	Survived	
21	2019	Pig butcher	/	Fever, headache, consciousness loss, seizures, bilateral retinal detachment, encephalitis	/	+	Survived	[80]
22	2020	Swineherder	/	Fever, coma, endophthalmitis	/	+	Survived	[81]
23	2021	Housewife	/	Fever, headache, seizures, coma, respiratory failure	/	+	Survived	[82]
24	2021	Swineherder	/		/	+	Died	
25	2021	Pig butcher	Hand injury at work	Fever, consciousness loss, seizures, respiratory failure	/	+	discharged with ventilator support	[83]

/ Data not provided in the reference. + Nucleotide sequences were detected positive in the cases.

Comparing the cases listed in Table 3 and Table 4, it seemed that the infectivity and infection characteristics of PRV in humans have significantly changed. The cases reported between 1914 and 1992 were diagnosed by clinical symptoms and contact history. The patients had contact with infected cats, dogs, or cattle and showed cold-like symptoms such as fever, sore throat, limb weakness, and itching in most cases (Table 3). With the development of detection technology, the diagnostic basis has become more detailed. The cases reported after 2017 were diagnosed by PRV-specific antibodies and nucleic acid. Infected pigs and contaminated pork were the common contact history. The patients generally started with influenza-like symptoms that quickly developed into neurological symptoms within five days, with some even dying or experiencing disability at the end of the disease (Table 4). Thence, the possible virus source in these cases and the virulence of the PRV strains might have changed. The PRV strains resulting in infection in patients have been reported to be phylogenetically closer to the PRV variant strains currently circulating in Chinese pig populations [70,74,76]. The variant strains isolated in China after 2012 have been sequenced and found to be quite different from foreign strains and Chinese classical strains such as Ea, Fa, LA, and SC. Based on genome sequencing of the variant stains TJ, HNX, and ZJ01 and a comparative analysis with the classical strains, VP1/2 (UL36), ICP22 (US1), and ICP4 (IE180) are the most variable proteins, and gE (US8), gB (UL27), gC (UL44), and gD (US6) are the main variable glycoproteins [84,85,86]. PRV *gE* is a crucial virulence factor related to the anterograde transport of viral particles in neurons [87] and is one of the genes commonly deleted in live attenuated vaccine strains. The experimentally constructed rLA-ZJ01/gEI developed by replacing the gE and gI of LA with the gE and gI of ZJ01 was more pathogenic to piglets than LA, implying that the changes in the gE and gI proteins partially contribute to the enhanced virulence of ZJ01 [86]. gB and gC are the core proteins required for the invasion of all herpesviruses and also the major immunogenic proteins [88]. PRV BJB that was reconstructed by replacing the gB of Bartha-K61 with the gB of JS-2012 showed increased protective efficacy against JS-2012 than Bartha-K61 [89]. Therefore, changes in these proteins are associated with the different biological characteristics of the PRV variant strains. It would be interesting to investigate the transmission and infection of PRV variant strains to humans based on these variations.

Additionally, to assess the risk of PRV infection in humans, it is vital to analyze whether all of the PRV variant strains or only specific PRV strains can infect humans. However, it is difficult to identify the general characteristics of PRV strains that infectious to humans since only one human-originated PRV strain has been isolated. In the phylogenetic analyses based on the *gE* and *gC* sequences of 54 PRV strains isolated from domestic pigs (44), dogs (9), and bovine (1) in Italy, most of the PRV strains from pigs, three of the strains from dogs working on pig farms, and PRV from bovine were closely related in the same clade, while five strains isolated from hunting dogs were highly close to the PRV strains from wild boars [90]. Therefore, it is presumed that the contact degree between different susceptible hosts was one of the critical points accounting for PRV cross-species transmission.

In fact, Nectin-1, Nectin-2, and HveD have been confirmed to mediate PRV infection in human and mouse cells [91]. Nectin-1 is highly conserved in mammals. Swine nectin-1 and human nectin-1 share 96% identity in amino acids, and they can both mediate the entry of HSV-1, herpes simplex virus-2 (HSV-2), PRV, and bovine herpesvirus 1 (BHV-1) [92,93]. PRV gD has been shown to bind swine nectin-1 and human nectin-1 with similar affinity, and the key residues of the interaction interface are conservative, providing structural evidence for PRV infection in humans [94]. However, there may be more ligands and receptors since it has been found that PRV can still infect Chinese hamster ovary cells, even without gD receptors [95]. PRV mutants without gD can be cultured and passaged to reach a high virus titer through cell-to-cell transmission [96]. Through porcine genome-wide CRISPR/Cas9 library screening, sphingomyelin synthase 1 (SMS1) was identified to be critical for PRV mutants without the *gD* gene to infect porcine kidney cells. When SMS1 was knocked out in the cells, the infection efficiency of PRV mutants without the *gD* gene decreased by 90%. This indicates that SMS1 plays a crucial role in PRV infection when the gD-mediated invasion pathway is blocked [97].

Moreover, HVEM mRNA and membrane-bound proteins have been shown to be expressed in the human adult retinal pigment epithelial cell line-19 (ARPE-19) [98], corneal fibroblasts cells [99], trabecular meshwork cells [100], conjunctival epithelial cells [101], and corneal epithelial cells [102]. Neutralizing antibodies or interfering RNA against HVEM could significantly reduce the entry of HSV-1 to these cells. Furthermore, previous studies have shown that HVEM can promote HSV-1 replication in mouse eyes [103,104]. Obviously, HVEM is associated with HSV-1 infection and pathogenicity in the eyes, so the common visual impairment in patients infected with PRV might be correlated to HVEM.

## 5. Vaccines and Diagnosis Methods for Pseudorabies

PRV infection in domestic pigs has been well controlled and even eliminated in many countries using vaccines and diagnostic tests, supporting the effectiveness of the DIVA concept. DIVA means the differentiation infected from vaccinated animals through the use of marker vaccines and respective serological diagnostic tests. After classically attenuated live vaccines developed by passaging, such as Bartha-K61, live virus vaccines lacking the major virulence-determining genes were developed by genetic engineering. The deletion of one or more genes targeting the *gE*, *gI*, *TK*, and *gG* genes are the typical choices [105,106,107,108]. Currently, based on homologous recombination, CRISPR/Cas9, bacterial artificial chromosome (BAC), and other genetic engineering technologies, gene-deletion strains can be rapidly constructed and assessed [108].

It should be noted that vaccine strains ought to be constructed based on the epizootic strains in the field to ensure the highest protection efficacy and reduce virus variation caused by recombination. Before 2011, pseudorabies in China had been well controlled by vaccination with Bartha-K61 and other vaccines constructed based on local classical strains. However, since late 2011, PRV variant strains have caused pseudorabies outbreaks in China. It has been reported that the variant strains are more virulent than the classical strains and that the classical vaccines can no longer provide sufficient protection against the variant strains [4,84]. The live attenuated vaccines based on variant strains such as SMX, TJ, ZJ01, and HN1201 were developed and showed adequate protection against the variant strains [107,109,110,111]. Therefore, it is necessary to monitor the changes of field strains continuously and to periodically construct new vaccine candidates. Field strains should be isolated from the wild boars in the countries in which pseudorabies has been eliminated in domestic pigs. Additionally, more detailed PRV typing methods are required to distinguish the differences among PRV strains and to select strains for vaccine development.

Meanwhile, diagnostic tests together with PRV gene-deletion vaccines are essential for applying DIVA. The indirect ELISA targeting of the gB and gE antibodies is one of the most widely applied serological approaches for differential diagnosis [5]. In addition, diverse molecular biological approaches targeting PRV genes have been established, such as PCR, real-time PCR, nano PCR, loop-mediated isothermal amplification (LAMP), and droplet digital PCR [108]. The sensitivity and specificity of these diagnostic approaches will undoubtedly be further improved. For areas with a low prevalence of pseudorabies, sensitivity is the primary concern of the diagnosis so that sporadically infected pigs can be diagnosed and eliminated. Timely diagnosis is vital for reducing the losses caused by the virus spreading among the pig population. Therefore, easy-to-operate, accurate, and on-site testing are required to develop new diagnostic methods. One of the difficulties among the current pseudorabies diagnostic technologies is that they cannot detect PRV during latency. In the final stage of pseudorabies eradication programs, infected pigs should be culled, while latently infected pigs cannot be detected using existing methods. They will be excluded through herd updating on the farms if there is no viral activation. However, many risk factors are associated with viral reactivation on pig farms. Therefore, developing specific methods to detect latently infected pigs is particularly important for the future prevention, elimination, and eradication of pseudorabies.

## 6. Conclusions

PRV is an important pathogen for pigs and other animals. As an alphaherpesvirus showing a relatively high rate of protein-coding variation, it is necessary to monitor the epidemiology and variations of this virus. In this review, we summarized PRV prevalence in China and worldwide, how PRV evolution was contributed to by natural selection and recombination, and PRV infections in animals and humans. All of this information facilitates future research and the control of pseudorabies. PRV elimination in the swine population should be further accelerated with better vaccines and diagnostic approaches. PRV can potentially infect humans, and further investigation is warranted.

## Figures and Tables

**Table 1:** The gE antibody positivity rate in different provinces in China.

Region	gE Positive Rate (gE Positive Samples/Total Samples)				Reference
	2016	2017	2018	2019	
Beijing	33.66% (662/1966)	/	20% (4/20)	/	[15,16]
Chongqing	1.6% (11/702)	9.4% (60/637)	7.5% (60/798)	11.5% (53/460)	[17]
Fujian	37.37% (111/297)	26.11% (53/203)	27.32% (50/183)	/	[18]
Guizhou	1.89% (27/1480)	16.85% (538/3192)	16.85% (538/3192)	8.5% (92/1078)	[19]
Guangdong	/	/	33.60% (1084/3226)	/	[16]
Guangxi	22.87% (854/3734)	23.71% (996/4200)	20.60% (766/3718)	/	[16,20]
Henan	26.21% (3513/13,404)	28.82% (4755/16,497)	25.31% (3000/11,854)	26.69% (3460/12,963)	[21]
Hebei	/	/	62.74% (367/585)	50.05% (5245/10,479)	[16,22]
Heilongjiang	15.36% (474/3086)	15.50% (539/3478)	11.64% (318/2731)	/	[23]
Hubei	/	/	13.21% (123/931)	/	[16]
Hunan	24.4% (344/1410)	23.2% (349/1504)	44.64% (1011/2265)	/	[24]
Jiangxi	40.1% (362/902)	34.6% (318/919)	27.41% (1769/6455)	/	[16,25]
Qinghai	28.17% (131/465)	19.75% (157/794)	/	/	[26]
Shandong	57.8% (2909/5033)	50.4% (2476/4915)	55.2% (2072/3753)	/	[27]
Sichuan	/	/	32.49% (952/2930)	/	[16]
Yunnan	/	/	17.07% (306/1793)	/	
Tianjin	40.43% (970/2399)	37.02% (2219/3793)	51.59% (1957/3793)	/	[28]

/ Data not provided in the reference.

**Table 2:** The reported recombination events of PRV.

Strain	Isolation Country	Recombination Pattern	Recombination Site	Reference
Yangsan	South Korean	genotype I and genotype II	*UL21*	[35]
FJ-W2, FJ-ZXF	Fujian, China	genotype I and genotype II	*gB*	[38]
FJ62	Sichuan, China	genotype I (Wild boar) and genotype II	*gB*	[43]
JSY13	Jiangsu, China	genotype I (Bartha) and genotype II (JSY7)	*UL42, UL19,* *UL18, UL10*	[44]
SC	China	genotype I (Bartha) and genotype II	*gC*	[45]
HeN1, Qihe547	China	genotype I and genotype II (vaccine strains)	/	[36]
SC, LA	China	genotype I and genotype II (early strains)	/	
ZJ01	China	genotype I and genotype II	/	

/ Data not provided in the reference. The gene names were shown in italics.

**Table 3:** Suspected case reports of human infection with PRV between 1914 and 1992.

Case	Year	Occupation	Contact History	Clinical Symptoms	Antibody Detection	Pathogen Detection	Outcome	Reference
1	1914	Lab technician	A laboratory cat with pseudorabies	Swelling, reddening, and intense itching of the wound and the surrounding area	/	/	Survived	[66]
2	1914	Lab technician			/	/	Survived	
3	1940	Lab technician	Got injured during contacting with a dog infected with PRV	Pruritus, erythema, pain, and aphthous stomatitis	/	/	Survived	[68]
4	1940	Lab technician			/	/	Survived	
5	1963	Animal handler	A dog infected with PRV following an outbreak of pseudorabies on a pig farm	Severe throat pain and weakness in the legs	/	/	Survived	[66]
6	1963	Animal handler			/	/	Survived	
7	1963	Veterinary			/	/	Survived	
8	1963	Nightwatchman			/	/	Survived	
9	1983	Tourist in Denmark	Indirect contact with a sick cat	Anorexia, weight loss, headache, arthralgia	Neutralizing antibody Titer: 1:8–1:16	/	Survived	[67]
10	1986	Tourist in France	Close contact with cats and other domestic animals	Dysphagia, experienced strange smells and taste		/		
11	1986	Tourist in France				/		
12–17	1992	Six workers on a cattle farm	Direct contact with PRV infected cattle	Pruritus of the palms that spread onto the arms and shoulders and lasted for several days	/	/	Survived	[69]

/ Data not provided in the reference.

## Data Availability

Not applicable.
